# Chronic Iron Deficiency Anemia as the Initial Manifestation of Undiagnosed Von Willebrand Disease in a Woman With Long-Standing Menorrhagia: A Case Report

**DOI:** 10.7759/cureus.108255

**Published:** 2026-05-04

**Authors:** Shamas Rafique, Iqra Rafiq

**Affiliations:** 1 Internal Medicine, BeeWell International Hospital, Islamabad, PAK; 2 Family Medicine, Family Medical Health Care PLLC, New York, USA; 3 Internal Medicine, First Affiliated Hospital of Xinjiang Medical University, Ürümqi, CHN; 4 General Medicine, Services Hospital Lahore, Lahore, PAK; 5 Medicine and Surgery, First Affiliated Hospital of Xinjiang Medical University, Ürümqi, CHN

**Keywords:** abnormal uterine bleeding, bleeding disorders, coagulation disorders, heavy menstrual bleeding, hematology, iron deficiency anemia, menorrhagia, von willebrand disease, vwf deficiency, women’s health

## Abstract

Iron deficiency anemia is a common clinical condition in reproductive‑age women and is frequently attributed to gynecologic blood loss. However, underlying inherited bleeding disorders remain underrecognized contributors. We present a case of a woman with chronic fatigue, weakness, and long-standing menorrhagia who was repeatedly treated for iron deficiency anemia without sustained improvement. Subsequent hematologic evaluation revealed Von Willebrand factor (VWF) deficiency consistent with Von Willebrand disease. This case highlights the importance of recognizing abnormal uterine bleeding as a potential manifestation of an underlying hemostatic disorder and underscores the need for early diagnostic evaluation to prevent prolonged morbidity and avoid delays in definitive management.

## Introduction

Von Willebrand disease (VWD) is the most common inherited bleeding disorder, with an estimated prevalence of up to 1% in the general population; however, clinically significant bleeding symptoms occur in approximately 0.01% [1]. It results from quantitative or qualitative abnormalities of Von Willebrand factor (VWF), a critical protein involved in platelet adhesion and stabilization of factor VIII. VWF activity, measured by the ristocetin cofactor assay, reflects the protein's functional ability to facilitate platelet plug formation at sites of vascular injury [2]. Among women, heavy menstrual bleeding is the most frequent presenting symptom and may be the earliest clinical manifestation of the disorder [3]. Despite this, VWD remains underdiagnosed, particularly in patients whose symptoms are attributed solely to gynecologic causes [4]. Chronic blood loss from heavy menstrual bleeding can result in persistent iron deficiency anemia, often leading to repeated but incomplete treatment with iron supplementation alone [5]. Recognition of this association is essential to ensure appropriate management, reduce diagnostic delay, and prevent long‑term complications, including severe anemia and impaired quality of life.

## Case presentation

A 32‑year‑old woman presented with progressive fatigue, generalized weakness, and reduced exercise tolerance over several years. She reported a history of heavy menstrual bleeding since menarche at age 12, lasting for 20 years. Her cycles were characterized by 7‑10 days of bleeding, passage of large clots, and frequent pad changes, often exceeding one pad every 1‑2 hours during peak flow. The calculated International Society on Thrombosis and Haemostasis Bleeding Assessment Tool (ISTH‑BAT) score was 6 (normal: <3 for women), with points allocated as follows: menstrual bleeding (scored 4: required regular iron therapy for anemia, interfered with daily activities, and resulted in pad changes every 1‑2 hours); no other bleeding symptoms (scored 0: epistaxis, cutaneous, dental, or surgical bleeding); and no family history of diagnosed bleeding disorder (scored 0, although symptomatic relatives were noted). Her symptoms had gradually worsened, significantly affecting her daily functioning and quality of life.

She had previously been diagnosed with iron deficiency anemia and treated intermittently with oral iron therapy (ferrous sulfate 325 mg daily and, at various times, ferrous gluconate 300 mg daily) for cumulative periods totaling approximately 18 months over five years, with only transient hemoglobin improvement (peak: 10.2 g/dL) and persistently low ferritin (never exceeding 15 ng/mL). No prior evaluation for a bleeding disorder had been performed. Family history was notable for heavy menstrual bleeding in a maternal aunt and the patient's mother (both requiring iron therapy, but no formal diagnoses), raising suspicion for an inherited bleeding diathesis.

On physical examination, the patient appeared pale, with mild tachycardia (heart rate: 102 bpm), but no evidence of active bleeding, petechiae, or ecchymosis. There was no hepatosplenomegaly or lymphadenopathy. Given the chronicity of symptoms and inadequate response to iron therapy, further evaluation for an underlying bleeding disorder was pursued.

Investigations

Initial laboratory evaluation demonstrated microcytic anemia and iron deficiency, as summarized in Table 1. The hemoglobin level of 8.9 g/dL confirmed moderate anemia, while the low mean corpuscular volume (70 fL) indicated microcytosis. A ferritin of 8 ng/mL, serum iron of 22 μg/dL, and transferrin saturation of only 5% established the diagnosis of absolute iron deficiency, with the elevated total iron‑binding capacity of 428 μg/dL reflecting compensatory increased transferrin production.

**Table 1:** Hematologic and Iron Studies Interpretation: Results confirm moderate microcytic anemia with absolute iron deficiency, evidenced by low ferritin, low serum iron, markedly reduced transferrin saturation (5%), and compensatory elevation of total iron‑binding capacity. MCV: mean corpuscular volume, TIBC: total iron-binding capacity, g/dL: grams per deciliter, fL: femtoliters, ng/mL: nanograms per milliliter, μg/dL: micrograms per deciliter, × 10³/μL: thousands per microliter

Parameter	Result	Reference range
Hemoglobin	8.9 g/dL	12-16 g/dL
MCV	70 fL	80-100 fL
Ferritin	8 ng/mL	15-150 ng/mL
Serum iron	22 μg/dL	50-170 μg/dL
TIBC	428 μg/dL	250-450 μg/dL
Transferrin saturation	5%	15%-50%
Platelet count	245 × 10³/μL	150-400 × 10³/μL

Further evaluation to exclude common non‑structural causes of abnormal uterine bleeding was performed, with results shown in Table 2. All endocrine studies were within normal limits: thyroid‑stimulating hormone was 2.1 mIU/L, prolactin was 14 ng/mL, and β‑human chorionic gonadotropin testing was negative, ruling out pregnancy.

**Table 2:** Endocrine and Gynecologic Evaluation Interpretation: All values are within normal limits, effectively excluding thyroid dysfunction, hyperprolactinemia, and pregnancy as contributors to abnormal uterine bleeding. TSH: thyroid-stimulating hormone, β-hCG: beta-human chorionic gonadotropin, mIU/L: milli-international units per liter, ng/mL: nanograms per milliliter

Test	Result	Reference range
TSH	2.1 mIU/L	0.5-5.0 mIU/L
Prolactin	14 ng/mL	5-25 ng/mL
β-hCG	Negative	Negative

**Figure 1:**
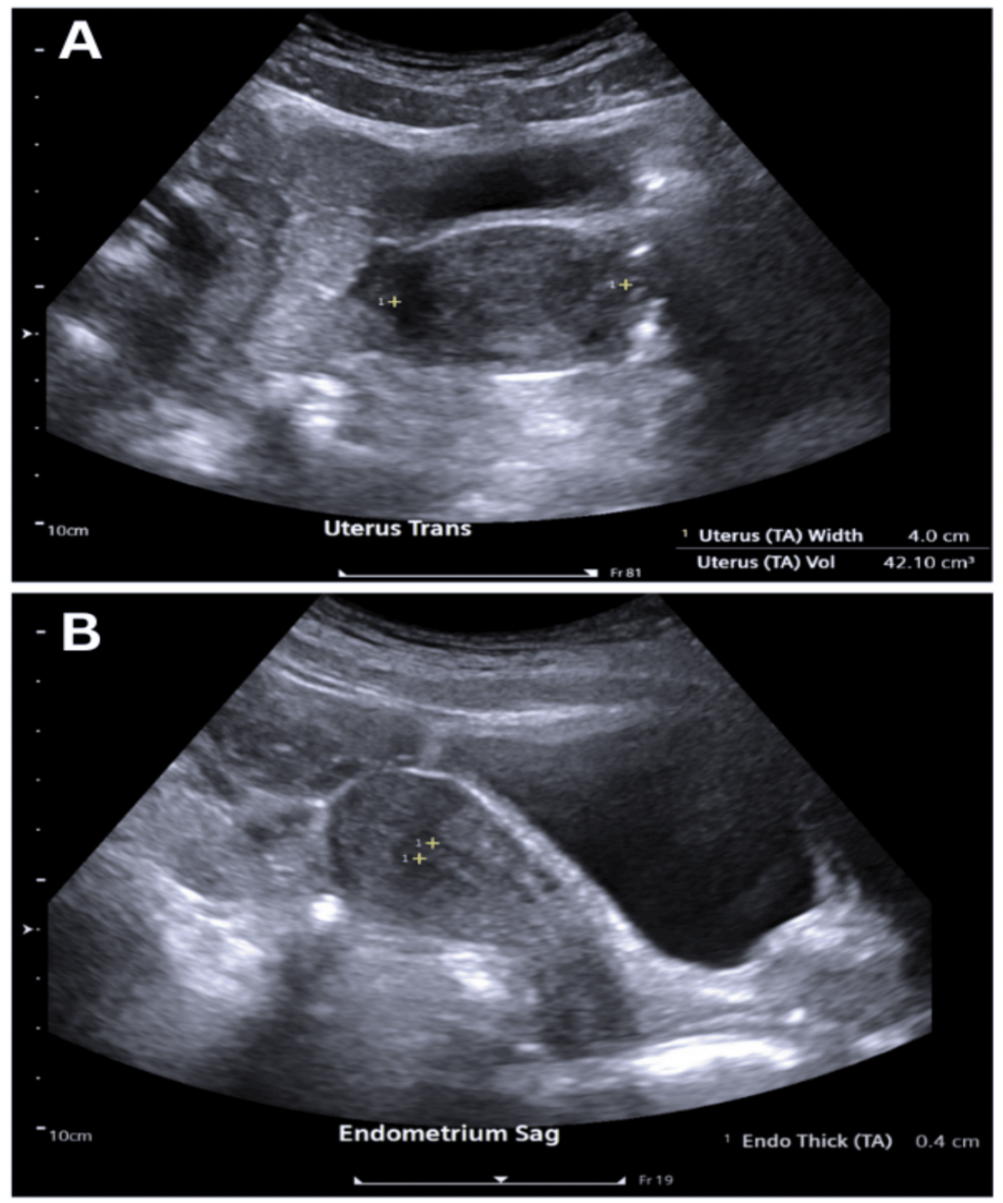
Pelvic ultrasound demonstrating normal uterine anatomy and endometrial thickness (A) Transverse view of the uterus showing normal contour and homogeneous myometrium. (B) Sagittal endometrial view showing thickness appropriate for menstrual phase without a focal intracavitary lesion. (Ultrasound performed on day 6 of the menstrual cycle; endometrial thickness measured as 0.4 cm (4 mm) as shown in Figure 1B.)

The pelvic ultrasound (Figure 1) was obtained on day 6 of the patient's menstrual cycle (early proliferative phase). It revealed a normal-sized anteverted uterus with homogeneous myometrial echotexture, endometrial thickness of 4 mm (appropriate for the early proliferative phase), and no evidence of fibroids, polyps, adenomyosis, or focal lesions; bilateral ovaries appeared normal with no adnexal masses or free fluid.

Given the clinical suspicion for an inherited bleeding disorder, a Von Willebrand panel was obtained, with results summarized in Table 3. The patient's ABO blood type was O positive. Notably, individuals with type O blood have baseline VWF levels approximately 25% lower than non-O individuals, which can contribute to lower levels; however, her values (VWF antigen: 35%, VWF activity: 30%) fall below the typical lower limit of normal even after accounting for this physiological variation. The Von Willebrand factor antigen level was reduced to 35% (reference range: 50%‑150%, lower limit of normal 50%). Von Willebrand factor activity (ristocetin cofactor) was similarly decreased to 30% (reference: 50%‑150%), yielding an activity/antigen ratio of 0.86 (preserved, indicating a quantitative rather than qualitative defect). Factor VIII activity was also modestly reduced to 45% (reference: 50%‑150%), consistent with impaired stabilization by VWF. These findings are diagnostic of type 1 Von Willebrand disease, although it is important to note that some guidelines classify VWF levels between 30% and 50% as "low VWF" rather than definitive VWD; however, our patient's activity level of exactly 30% falls at the diagnostic threshold, and repeat testing confirmed persistence, supporting the diagnosis. Repeat testing at a separate time point (two weeks later) confirmed persistently reduced VWF levels (VWF antigen: 33%, VWF activity: 28%), minimizing the possibility of transient suppression due to acute illness, stress, or hormonal fluctuations.

**Table 3:** Coagulation and VWF profile Interpretation: Reduced VWF antigen (35%) and activity (30%) with a preserved activity/antigen ratio of 0.86 confirms a quantitative deficiency consistent with type 1 VWD. The mild reduction in factor VIII activity (45%) is secondary to impaired VWF-mediated stabilization. VWF: Von Willebrand factor, RCo: ristocetin cofactor, FVIII: factor VIII, percent (%) of normal activity/antigen level Note: The activity/antigen ratio is a diagnostic calculation. A ratio >0.7 indicates a quantitative deficiency (type 1 VWD), while a ratio <0.7 suggests a qualitative defect (type 2 VWD).

Test	Result	Reference range
VWF antigen	35%	50%-150%
VWF activity (RCo)	30%	50%-150%
Factor VIII activity	45%	50%-150%
Activity/antigen ratio	0.86	Preserved (>0.7), consistent with type 1 VWD

Diagnostic considerations and clinical reasoning

The patient's long-standing menorrhagia since menarche, family history of similar symptoms, and persistent iron deficiency anemia despite cumulative oral iron therapy over 18 months raised a strong suspicion for an inherited bleeding disorder. The combination of reduced VWF antigen and activity with a preserved activity/antigen ratio (0.86) supported a quantitative defect, consistent with type 1 VWD. A mild reduction in factor VIII further supported impaired VWF‑mediated stabilization. Alternative causes of abnormal uterine bleeding, including structural gynecologic pathology and endocrine abnormalities (thyroid dysfunction and hyperprolactinemia), were excluded based on imaging and laboratory findings. The absence of thrombocytopenia or other hematologic abnormalities further supported a primary VWF‑related defect.

Management and outcome

The therapeutic approach and clinical rationale are summarized in Table 4.

**Table 4:** Therapeutic interventions DDAVP: desmopressin (1-deamino-8-D-arginine vasopressin), VWF: Von Willebrand factor

Intervention	Clinical rationale
Oral iron	Replete stores, correct anemia
Tranexamic acid + hormonal therapy	Reduce menstrual blood loss (antifibrinolytic + cycle regulation)
Desmopressin (DDAVP)	Induce endogenous VWF release
VWF concentrate (if needed)	Replacement for refractory cases

The patient was treated with iron supplementation (ferrous sulfate 325 mg daily), antifibrinolytic therapy (tranexamic acid 1300 mg three times daily) during menses, and combined oral contraceptive therapy for menstrual regulation. A desmopressin (DDAVP) challenge was administered at a dose of 0.3 μg/kg intranasally. Baseline levels were as follows: VWF antigen, 35%; VWF activity, 30%; and factor VIII, 45%. One hour after administration, peak levels were as follows: VWF antigen, 82% (normalized); VWF activity, 72% (normalized); and factor VIII, 110% (normalized). At four hours, levels remained elevated: VWF antigen, 65%; VWF activity, 58%; and factor VIII, 95%. This response confirmed adequate endogenous VWF release and ruled out an accelerated clearance phenotype (e.g., type 1C VWD). During follow-up, the patient reported marked improvement in symptoms, with a reduction in menstrual bleeding and progressive normalization of hemoglobin (12.1 g/dL at six months) and ferritin (42 ng/mL) levels. Notably, despite the severity of her iron deficiency (ferritin: 8 ng/mL, transferrin saturation: 5%), intravenous iron was discussed but deferred because the combination of tranexamic acid and hormonal therapy rapidly reduced menstrual blood loss, allowing oral iron to become effective without the need for IV access or its associated risks. This approach was successful, as demonstrated by the normalization of her hemoglobin and ferritin within six months. No bleeding complications were reported during the follow‑up period.

## Discussion

This case highlights a common yet underrecognized scenario: an inherited bleeding disorder presenting as chronic iron deficiency anemia from long-standing menorrhagia. Heavy menstrual bleeding occurs in 60%‑90% of women with VWD and is often the earliest symptom [3,6]. Diagnostic delay is common, partly because affected families normalize heavy bleeding, as seen here. In a retrospective cohort study, women with VWD experienced a median diagnostic delay of 14.2 years from the first bleeding event, with 40% having at least three bleeding episodes before diagnosis [1]. Similarly, a recent review noted an average diagnostic delay of 8‑16 years for women with inherited bleeding disorders, largely due to insufficient awareness of gynecologic symptoms among healthcare professionals [7]. In our patient, the 20‑year interval from menarche to diagnosis illustrates these persistent systemic gaps.

Type 1 VWD accounts for 70%‑80% of cases and is diagnosed by reduced VWF antigen and activity with a preserved ratio [1,7]. VWF levels fluctuate with stress, hormones, and estrogen, necessitating repeat testing when suspicion is high. The diagnostic distinction between "low VWF" (levels 30‑50 IU/dL) and definitive type 1 VWD (levels <30 IU/dL) remains debated; current American Society of Hematology (ASH), International Society on Thrombosis and Haemostasis (ISTH), National Hemophilia Foundation (NHF), and World Federation of Hemophilia (WFH) guidelines recommend classifying patients with VWF levels of 30‑50 IU/dL and an increased bleeding phenotype as type 1 VWD [8]. Our patient's VWF activity of 30%, symptomatic menorrhagia, family history, and persistently low levels on repeat testing support the diagnosis of type 1 VWD. Even if classified as "low VWF," such patients benefit from hemostatic therapy [9].

International guidelines recommend bleeding disorder workup for heavy menstrual bleeding, especially when anemia, family history, or poor treatment response is present [1,4]. A ferritin threshold of <30 ng/mL captures iron deficiency before anemia develops and should prompt evaluation [10]. Early diagnosis enables targeted therapies (antifibrinolytics, hormonal therapy, and desmopressin), which reduce bleeding and improve quality of life [7,9]. Failure to diagnose VWD increases surgical, obstetric, and trauma risks [11]. A multidisciplinary approach (hematology and gynecology) is essential.

Our case mirrors previously reported experiences of prolonged diagnostic delay in women with VWD. Brignardello-Petersen et al., in a systematic review of gynecologic and obstetric management of VWD, described women in whom recurrent menorrhagia and iron deficiency anemia persisted for years before hematologic evaluation, often because normal pelvic imaging was interpreted as excluding a pathologic cause of bleeding [11]. Similarly, our patient's 20‑year latency from menarche to diagnosis, during which she received only episodic oral iron therapy, underscores the same systemic failure to pursue coagulation testing when structural gynecologic pathology has been excluded.

This case underscores that iron deficiency anemia is not a diagnosis in itself but a symptom of an underlying pathology. In the presence of long-standing menorrhagia, a normal pelvic ultrasound should not be the end of the workup but rather the trigger to investigate the coagulation cascade.

## Conclusions

This case demonstrates that Von Willebrand disease should be considered in women with chronic menorrhagia and refractory iron deficiency anemia. Systematic evaluation from identifying microcytic anemia with ferritin 8 ng/mL, excluding structural and endocrine causes, to confirming reduced VWF antigen (35%) and activity (30%) with a preserved ratio provides a reproducible diagnostic framework. The patient's blood type O positive and robust DDAVP response (peak VWF activity: 72%) further support the diagnosis and therapeutic strategy.

For women with unexplained menorrhagia and persistent iron deficiency despite oral iron, a bleeding disorder should be suspected even without other bleeding manifestations. Routine family history and a low threshold for Von Willebrand testing can identify previously undiagnosed cases. Timely diagnosis alleviates chronic anemia and menstrual morbidity while enabling proactive planning for hemostatic challenges, including pregnancy, surgery, and trauma.
